# Supplementary material for: Photos provide information on age, but not kinship, of Andean bear
Source: PeerJ. 2015 Jul 16;3:e1042. doi: 10.7717/peerj.1042 (PMC4512767; doi:10.7717/peerj.1042)
Supplement: Supplemental Information 1 [file peerj-03-1042-s001.pdf]

INSTITUTIONAL ANIMAL CARE AND USE COMMITTEE
ZOOLOGICAL SOCIETY OF SAN DIEGO

MEMORANDUM

To: Russ Van Horn, Robyn Appleton, Ron Swaisgood

From: Rebecca Papendick, IACUC Chair RP

Date: November 15, 2010

Re: IACUC Proposal #10-023

. ■ . ■ . ■ . ■ . ■ . ■ . ■ . ■ . ■ . ■ . ■ . ■ . ■ . ■ .

This is to inform you that the IACUC approved Proposal #10-023, "Behavioral Ecology and Conservation of Andean (spectacled) bears in Peru".

The Zoological Society of San Diego has an Animal Welfare Assurance on file with the Office of Laboratory Animal Welfare (OLAW). The Assurance Number is A3675-01.
