# Supplementary material for: Photos provide information on age, but not kinship, of Andean bear
Source: PeerJ. 2015 Jul 16;3:e1042. doi: 10.7717/peerj.1042 (PMC4512767; doi:10.7717/peerj.1042)
Supplement: Supplemental Information 2 [file peerj-03-1042-s002.pdf]

REPUBLICA DEL PERU

DIRECCIÓN GENERAL FORESTAL Y DE FAUNA SILVESTRE
COPIA FIEL DEL ORIGINAL
DOY FÉ
FEDATARIA TITULAR
IRMA IRAIDA BRICEÑO SANCHEZ
DGFFS

RESOLUCIÓN DIRECTORAL N°0245-2012-AG-DGFFS-DGEFFS

Lima, 18 JUL. 2012

VISTA:

La solicitud de Autorización para realizar investigación científica fuera de Áreas Naturales Protegidas con colecta de flora y/o fauna silvestre por el período de hasta tres años con código único de trámite N° 4768 de fecha de recepción 16 de enero de 2012, presentada por la señorita Robyn Appleton identificada con Pasaporte N° WD122220, y el Informe Técnico Legal N° 2365-2012-AG-DGFFS-DGEFFS, y;

CONSIDERANDO:

Que, Mediante solicitud de fecha de recepción 16 de enero de 2012, la señorita Robyn Appleton, investigadora de Spectacled Bear Conservation Society (SBC) , solicitó autorización para realizar actividades de investigación científica de flora y/o fauna silvestre con un grupo de investigadores, fuera de Áreas Naturales Protegidas en el Parque Arqueológico y Ecológico de Batan Grande en el departamento de Lambayeque, para desarrollar el proyecto "Ecología y abundancia de osos andinos en los bosques secos del noroeste de Perú: Pozas de agua como oportunidades de investigación y desafíos en la conservación", por el período de tres (03) años;

Que, Mediante carta de fecha 25 de enero de 2012, el señor Carlos Elera Arévalo director del Museo Nacional de Sicán, solicita a esta dirección que se le conceda el permiso correspondiente a la señorita Robyn Appleton para realizar dicha investigación, en el ámbito del Parque Arqueológico y Ecológico de Batan Grande;

Que, mediante Carta N° 246-2012-AG-DGFFS-DGEFFS del 21 de febrero de 2012, emitida por la Dirección de Gestión Forestal y de Fauna Silvestre, se solicitó a la señorita Robyn Appleton, remitir el plan de investigación según formato adjunto, el curriculum vitae del investigador responsable y las cartas de presentación originales de los investigadores participantes. Asimismo, debido a que el estudio se realizará en el ámbito territorial de comunidades campesinas, se solicito a la usuaria la Carta de Autorización de la Comunidad Campesina "Santa Lucia". Por otro lado, considerando que el estudio involucra la captura de individuos, se solicitó a la usuaria, remitir el CV del veterinario especializado en fauna silvestre que participará en el proyecto, así como la opinión de un especialista de una institución reconocida por la comunidad científica, con experiencia en el grupo de estudio, en la que se indique que no se incrementará el riesgo del estado de conservación de la especie y sus poblaciones categorizadas como En Peligro Crítico y En Peligro. Finalmente, se indicó a la usuaria de contemplar la futura exportación de muestras de los especímenes incluidos en la

DIRECCIÓN GENERAL FORESTAL Y DE FAUNA SILVESTRE
COPIA FIEL DEL ORIGINAL
DOY FÉ
FEDATARIA TITULAR
IRMA IRAIDA BRICEÑO SÁNCHEZ
DGFFS

CITES, será necesario la opinión favorable de la Autoridad Científica CITES Perú correspondiente;

Que, mediante carta de fecha 26 de abril de 2012, el Ph.D. Allison Alberts, Directora del Instituto de Conservación e Investigación del San Diego Zoo Global, remite a esta dirección la carta de presentación del señor Rusell. C. Van Horn, investigador del Instituto de Conservación e Investigación del San Diego Zoo Global, quien participará en dicho proyecto de investigación;

Que, mediante carta de fecha 26 de abril de 2012, la Blga. Jessica Amanzo, remite a esta dirección la carta de presentación de los investigadores participantes;

Que, mediante carta de fecha 21 de mayo de 2012, la señorita Robyn Appleton, investigadora principal del proyecto, remite a esta dirección el plan de investigación según formato, curriculum vitae de la investigadora responsable y la carta de presentación de dos participantes;

Que, mediante carta de fecha 01 de junio de 2012, la señorita Robyn Appleton, remite a esta dirección las copias de las denuncias archivadas e infundadas hacia de la investigadora responsable y su grupo de investigadores por parte de La Cordinadora Nacional de Rondas Campesinas, las Comunidades Campesinas de Tocmoche, Santa Lucía de Ferreñafe, y la Sociedad Zoologica de Frankfurt, por la Primera Fiscalía Provincial Penal Corporativa de Ferrañefe, la Cuarta Fiscalía Superior Penal Transitoria de Lambayeque y la Segunda Fiscalía Superior Penal de Liquidación de Lambayeque respectivamente. Asimismo, se presentan dos copias de las querellas presentadas en respuesta a estas denuncias. Por otro lado, se remite una copia fedateada de la carta de Permiso de la Comunidad Campesina Santa Lucía de Ferreñafe de fecha 30 de noviembre de 2011. No obstante, la usuaria señala que hasta la fecha de la emisión de la presente carta, la SUNARP no registra la directiva legítima de esta comunidad, debido a una apelación. Por último, la usuaria adjunta a la presente carta, su certificación para realizar inmovilizaciones sin veterinario otorgada por el Comité Institucional de Cuidado y Uso Animal del Zoológico de San Diego, valida hasta el 2013, así como la carta que avala su experiencia en inmovilizaciones de osos andinos y el curriculum vitae de la MV. PhD. Meg Sutherland-Smith, participante del presente proyecto;

Que, mediante Carta N° 682-2012-AG-DGFFS-DGEFFS de fecha 06 de junio de 2012, se solicita a la usuaria remitir la siguiente documentación: a) la Carta de Autorización de ingreso a la comunidad campesina de Santa Lucía firmada por su presidente, quien debe estar facultado para tal fin, con poderes inscritos en Registros Públicos; b) La participación de un médico veterinario en el procedimiento de contención química, sedación y anestesia, hasta la recuperación total del individuo; c) la opinión actualizada y en original de una institución reconocida por la comunidad científica nacional o internacional, con experiencia en el grupo de estudio, en la que se indique que no se incrementará el riesgo de estado de conservación de las poblaciones de la especie objeto de estudio categorizada como En Peligro según el Decreto Supremo 034-2004-AG que aprueba la categorización de especies amenazadas d) Remitir la opinión favorable de la Autoridad Científica CITES-Perú correspondiente, si el proyecto requerirá la futura exportación de la especies incluida en el Apéndice I de la CITES;

REPUBLICA DEL PERU

DIRECCIÓN GENERAL FORESTAL Y DE FAUNA SILVESTRE
COPIA FIEL DEL ORIGINAL
DOY FÉ
FEDATARIA TITULAR
IRMA IRAIDA BRICEÑO SÁNCHEZ
DGFFS

Que, mediante Carta de fecha 25 de junio de 2012, la señorita Robyn Appleton, remite a esta dirección: a) la carta de presentación del veterinario que participará y supervisará los procedimientos de cada inmovilización de individuos de oso andino y b) la opinión del especialista con experiencia en el grupo de estudio, que a su vez es una autoridad científica CITES. Finalmente, la usuaria señala que en relación a la carta de autorización de la comunidad, el área de estudio se encuentra fuera de la Comunidad Campesina Santa Lucía de Ferreñafe y por el momento se utilizarán únicamente los accesos fuera de los territorios comunales, hasta que la nueva directiva esté inscrita en la SUNARP, y se solicité el tramite respectivo para el acceso;

Que, el Decreto Supremo Nº 014-2001-AG, Reglamento de la Ley Forestal y de Fauna Silvestre, establece en el artículo 328° que la investigación científica o estudio que implique colección de especímenes o elementos de la flora y fauna silvestre no vedados y la obtención de datos e información de campo, requiere autorización del INRENA;

Que, la Tercera Disposición Final del Decreto Supremo Nº 003-2009-MINAM, que eleva a rango de Decreto Supremo a la Resolución Ministerial Nº 087-2008-MINAM y ratifica la aprobación del Reglamento de Acceso a los Recursos Genéticos, indica que la obtención de permisos, autorizaciones y demás documentos que otorguen entidades públicas, tales como el Ministerio de Agricultura y que amparen la investigación, obtención, provisión, transferencia u otro de recursos biológicos, con fines distintos a su utilización como fuente de recursos genéticos, no faculta a sus titulares a utilizar dichos recursos como medio para acceder a los recursos genéticos, ni determinan ni presumen autorización de acceso;

Que, la Resolución Ministerial Nº 212-2011-AG que aprueba el Texto Único de Procedimientos Administrativos (TUPA) del Ministerio de Agricultura, establece en su numeral 21, los requisitos para la Autorización para realizar actividades de investigación científica y filmaciones con fines comerciales de flora y fauna silvestre fuera de Áreas Naturales Protegidas;

Que, el Informe Técnico Legal Nº 2365-2012-AG-DGFFS-DGEFFS del 12 de julio del presente año, la Dirección de Gestión Forestal y de Fauna Silvestre de la Dirección General Forestal y de Fauna Silvestre, concluye que el presente estudio reviste de importancia pues a comprender la distribución actual, ecología y etología de los osos andinos *Tremarctos ornatus*, estimar el tamaño poblacional en los bosques secos, identificar los aspectos críticos de su hábitat;

Que, el referido informe señala que el proyecto cuenta con la participación de renombrados especialistas en fauna silvestre tanto nacionales, como extranjeros. Asimismo, en el proceso de sedación, anestesia y toma de muestras, se contará con la participación de la médico veterinario Meg Sutherland-Smith de la Sociedad Zoológica de San Diego, que posee amplia experiencia en fauna silvestre y en procedimientos asociados a la inmovilización de osos silvestres y en cautiverio. Dichos protocolos han sido revisados y aprobados por el Comité Institucional de Uso y Cuidado de los Animales (IACUC) del Zoológico de San Diego. Finalmente, el proceso de inmovilización cuenta con la asistencia de la MSc. Robyn Appleton

DIRECCIÓN GENERAL FORESTAL Y DE FAUNA SILVESTRE · DGFFS ·
COPIA FIEL DEL ORIGINAL
DOY FÉ
FEDATARIA TITULAR
IRMA IRAIDA BRICEÑO SANCHEZ

de la Spectacled Bear Conservation Society (SBC) y el PhD. Russell C. Van Horn del Instituto de Conservación e Investigación de la Sociedad Zoológica de San Diego, los cuales han sido certificados para esta actividad debido a su experiencia en proyectos con osos silvestres y entrenamiento adicional en San Diego;

Que, el mencionado informe indica que presente estudio cuenta con la opinión favorable del PhD Víctor Pacheco, Jefe del Departamento de Mastozoología del Museo de Historia Natural de la Universidad Nacional Mayor de San Marcos y profesional experto en grupos taxonómicos de las especies incluidas en los Apéndices de la CITES y acreditado para ejercer la función de Autoridad Científica CITES – Perú, quien expresa su conformidad a los métodos y protocolos utilizados para realizar el mencionado proyecto ya que no transgreden las normas de las convención CITES, ni representan ningún peligro para las poblaciones de osos de anteojos *Tremarctos ornatus*;

Que, el referido Informe indica que la presente propuesta forma parte de un proyecto a largo plazo, que ha cumplido con las recomendaciones de esta Dirección, presentación de informes de los años anteriores y de resultados, además se ha cumplido con el compromiso de capacitar a investigadores peruanos. Finalmente, se indica que la presenta investigación no se realizará en el ámbito territorial de comunidades campesinas no autorizadas;

Que, el mencionado Informe recomienda, brindar la autorización solicitada para la investigación científica, la captura temporal para la puesta de collar para monitoreo por radiotelemetría y la colecta de muestras de muestras de pelo, heces, sangre y parásitos de *Tremarctos ornatus* y la colecta de heces de *Tremarctos ornatus* sin captura. Asimismo, se considera procedente autorizar la colecta de las especies de moluscos (Clase Mollusca) y la colecta de frutos, tallos y hojas de las especies de flora que conforman la dieta de *Tremarctos ornatus*;

En uso de las atribuciones conferidas por el artículo 61º del Decreto Supremo Nº 031-2008-AG, que aprueba el Reglamento de Organización y Funciones del Ministerio de Agricultura que en su inciso n) precisa como funciones de la Dirección de Gestión Forestal y de Fauna Silvestre la de autorizar la extracción de especímenes de flora, fauna silvestres y microorganismos con fines de investigación;

SE RESUELVE:

Artículo 1º.- Autorizar a Robyn Appleton, la investigación científica en flora y fauna silvestre, la captura temporal para la puesta de collar para monitoreo por radiotelemetría y la colecta de muestras de muestras de pelo, heces, sangre y parásitos de *Tremarctos ornatus*, la colecta de heces de *Tremarctos ornatus* sin captura de acuerdo al Anexo 1 de la presente Resolución y la colecta de las especies de moluscos (Clase Mollusca) y la colecta de frutos, tallos y hojas de las especies de flora que conforman la dieta de *Tremarctos ornatus*, fuera de Áreas Naturales Protegidas en el departamento Lambayeque, en las localidades de Batan Grande y Mochumi Viejo, como parte del proyecto titulado "Ecología y abundancia de osos andinos en los bosques secos del noroeste de Perú: Pozas de agua como oportunidades de

REPUBLICA DEL PERU

DIRECCIÓN GENERAL FORESTAL Y DE FAUNA SILVESTRE
COPIA FIEL DEL ORIGINAL
DOY FE
FEDATARIA TITULAR
IRMA IRAIDA BRICEÑO SÁNCHEZ
DGFFS

investigación y desafíos en la conservación "; por el período de tres (03) años, contado a partir de la emisión de la presente Resolución Directoral. En la ejecución de la presente Resolución participarán los siguientes investigadores:

ROBYN APPLETON	PAS Nº WD122220
JESSICA AMANZO ALCANTARA	DNI N° 10064903
DANIEL JOSE VALLEJOS AGUINAGA	DNI N° 42427336
MEG SUTHERLAND SMITH	PAS N° 207811175
RUSSELL C. VAN HORN	PAS N° 458556154
FERNANDO RAUL ANGULO PRATOLONGO	DNI N° 10220996
ALVARO GARCIA OLAECHEA	DNI N° 44490711
ROMER ISAÍ SANCHEZ AGUINAGA	DNI N° 41857633
JAVIER VALLEJOS GUERRERO	DNI N° 28103153

Artículo 2º.- Los investigadores autorizados se comprometen a:

a) Colectar únicamente especímenes de especies autorizadas con fines científicos.
b) No ceder el material colectado a terceros.
c) Si por razones científicas acotadas, se requiere enviar al extranjero parte del material colectado, los interesados deberán gestionar el correspondiente Permiso de Exportación ante la Dirección General Forestal y de Fauna Silvestre, así como pasar el control respectivo.
d) Entregar el 50% del material colectado por tipo de muestra a una institución científica nacional debidamente reconocida. Los ejemplares únicos de los grupos taxonómicos colectados y holotipos, sólo podrán ser exportados en calidad de préstamo.
e) No contactar, ni ingresar a los territorios comunales sin contar con la autorización de las autoridades comunales correspondientes.
f) Notificar a la Dirección General Forestal y de Fauna Silvestre, en un plazo no mayor de 72 horas, por vía electrónica o telefónica, la captura de cada individuo de *Tremarctos ornatus* para la toma de muestras y la puesta de collar para monitoreo por radiotelemetría o de cualquier incidencia de mortalidad.
g) Remitir a la Dirección General Forestal y de Fauna Silvestre un reporte escrito, en relación a cada captura que se realice durante el desarrollo del proyecto, así como un reporte escrito de cualquier incidencia de mortalidad, en un plazo no mayor a los 15 días, incorporando la información digital de las cámaras trampa y filmación de la captura y liberación del individuo.
h) Remitir a Dirección General Forestal y de Fauna Silvestre informes semestrales de las principales actividades, avances, logros, dificultades, recomendaciones del mencionado proyecto,
i) Las capturas y protocolos de sedación se realizarán o serán asistidas únicamente por veterinarios especialistas en fauna silvestre de comprobada experiencia.
j) Los co investigadores nacionales desarrollarán actividades propias de su especialidad en todas las etapas del proyecto y velarán para que el desarrollo de la investigación se ajuste al plan de investigación aprobado.
k) Entregar a la Dirección General Forestal y de Fauna Silvestre dos (02) copias del informe final en idioma español, como resultado de la autorización otorgada, copias del material fotográfico y/o slides que puedan ser utilizadas para difusión. Así mismo entregar

CERTIFICO
Que la presente Fotocopia es auténtica y Exactamente igual al documento original que he tenido a la vista y con el cual ha sido Confrontada.
Lima 18 JUL 2012
IRMA IRAIDA BRICEÑO SÁNCHEZ
FEDATARIA TITULAR
R.M. Nº 0222-2011-AG

tres (03) copias de las publicaciones, producto de la investigación realizada en formato impreso y digital, que incluya la lista taxonómica de las especies de fauna y flora objeto de la presente autorización de colecta con las respectivas coordenadas (en formato excel).

l) Indicar el número de la Resolución en las publicaciones generadas a partir de la autorización concedida.

Artículo 3º.- La Dirección de Gestión Forestal y de Fauna Silvestre no se responsabiliza por accidentes o daños sufridos por los solicitantes de esta autorización, durante la ejecución del proyecto; asimismo, se reserva el derecho de demandar del proyecto de investigación los cambios a que hubiese lugar en los casos en que se dicten nuevas disposiciones legales o se formulen ajustes sobre la presente autorización.

Artículo 4º.- Los derechos otorgados sobre los recursos biológicos no otorgan derechos sobre los recursos genéticos contenidos en ellos. La presente no faculta el acceso a los recursos genéticos.

Artículo 5º.- El incumplimiento de los compromisos adquiridos será causal para denegar futuras autorizaciones a nivel institucional.

Artículo 6º.- Notificar la presente resolución a la señorita Robyn Appleton y transcribirla a la Dirección de Información y Control Forestal y de Fauna Silvestre y a la Administración Técnica Forestal y de Fauna Silvestre de Lambayeque.

Regístrese y comuníquese

Abog. Silvia Velásquez Silva
Directora de Gestión Forestal y de Fauna Silvestre (e)

REPUBLICA DEL PERU

DIRECCIÓN GENERAL FORESTAL Y DE FAUNA SILVESTRE - DGFFS
COPIA FIEL DEL ORIGINAL
DOY FE
FEDATARIA TITULAR
IRMA IRAIDA BRICEÑO SÁNCHEZ

ANEXO 1

Asimismo, la **captura temporal** para puesta de collar para monitoreo por radiotelemetría y extracción de muestras biológicas (sangre, suero, heces, pelos y tejido) de acuerdo al siguiente detalle:

**Taxa**	**Método de Colecta**	**N° de muestras**	**Tipo de muestra**
Tremarctos ornatus	Captura, colecta y liberación	5	Pelos, heces, saliva, parásitos y sangre y para puesta de collar
Tremarctos ornatus	No invasiva / sin captura	50	Heces
