# Supplementary material for: Photos provide information on age, but not kinship, of Andean bear
Source: PeerJ. 2015 Jul 16;3:e1042. doi: 10.7717/peerj.1042 (PMC4512767; doi:10.7717/peerj.1042)
Supplement: Supplemental Information 3 [file peerj-03-1042-s003.pdf]

MIAMI UNIVERSITY

Research Compliance Office
102 Roudebush Hall
Miami University, Oxford, OH 45056

2-Apr-15

To: Russell Van Horn (rvanhorn@sandiegozoo.org)

RE: Does perceived similarity of markings among Andean bears reflects the bears kinship?

Project reference number is: 01632e
(please refer to this ID number in all correspondence to compliance administration)

The project noted above and as described in your application for registering Human Subjects (HS) research has been screened to determine if it is regulated research or meets the criteria of one of the categories of research that can be exempt from approval of an Institutional Review Board (per 45 CFR 46). The determination for your research is indicated below.

The research described in the application is regulated human subjects research, however, the description meets the criteria of at least one exempt category included in 45 CFR 46 and associated guidance.

The Applicable Exempt Category(ies) is/are: 2

Research may proceed upon receipt of this certification and compliance with any conditions described in the accompanying email message. When research is deemed exempt from IRB review, it is the responsibility of the researcher listed above to ensure that all future persons not listed on the filed application who i) will aid in collecting data or, ii) will have access to data with subject identifying information, meet the training requirements (CITI Online Training).

If you are considering any changes in this research that may alter the level of risk or wish to include a vulnerable population (e.g. subjects <18 years of age) that was not previously specified in the application, you must consult the Research Compliance Office before implementing these changes.
Exemption certification is not transferrable; this certificate only applies to the researcher specified above. All research exempted from IRB review is subject to post-certification monitoring and audit by the compliance office.

Neal H. Sullivan, PhD
Director of Research Compliance
Miami University
Oxford, OH 45056
neal.sullivan@MiamiOH.edu
(513) 529-2488

Note: Approval is based on a post-hoc review of risk and procedures. Based on the description, this project has been determined to qualify for exemption from IRB oversight.
